# Supplementary material for: Transforming Growth Factor-β1 Selectively Recruits microRNAs to the RNA-Induced Silencing Complex and Degrades CFTR mRNA under Permissive Conditions in Human Bronchial Epithelial Cells
Source: Int J Mol Sci. 2019 Oct 5;20(19):4933. doi: 10.3390/ijms20194933 (PMC6801718; doi:10.3390/ijms20194933)
Supplement: Supplementary file 1 [file ijms-20-04933-s001.pdf]

TABLE S1. Small RNA-*seq* in WT- and F508del-CFBE cells showing those miRNAs upregulated (A) or downregulated (B) by TGF-β1 that are predicted to target human CFTR 3′UTR. These miRNAs are marked with black circles in the volcano plots in Figure 8. Only miRNAs with -log10 (P value) > 1.6 ($p < 0.025$) are listed. The miRNAs uniquely dysregulated in WT- or F508del-CFBE cells are in bold. Three miRNAs in italics are not shown in Figure 8 due to the plot cut-off area. Sequences of miRNAs were extracted from miRBase database and were used in miRmap database to search their predicted binding in 3′UTR of CFTR mRNA. *, $p < 0.05$; **, $p < 0.01$; ***, $p < 0.001$; ****, $p < 0.0001$.

A. Upregulated miRNAs					
**WT-CFBE Cells**	**Log2FC**	**-log10 (P Value)**	**F508del-CFBE Cells**	**Log2FC**	**-log10 (P Value)**
**hsa-miR-3168****	2.459	2.844	*hsa-miR-100-3p*****	1.772	15.686
*hsa-miR-143-3p*****	2.339	53.788	**hsa-miR-371b-3p******	1.464	6.016
**hsa-miR-3150b-3p***	2.297	1.787	*hsa-miR-181a-3p*****	1.283	7.659
**hsa-miR-8071*****	2.064	3.436	*hsa-miR-143-3p*****	1.224	8.421
**hsa-miR-152-5p****	1.680	2.715	hsa-miR-1185-1-3p*	1.078	1.749
**hsa-miR-1266-5p****	1.661	2.393	**hsa-miR-548h-3p******	1.053	4.629
**hsa-miR-625-3p***	1.432	1.773	hsa-miR-485-3p***	0.941	3.962
**hsa-miR-3924****	0.992	2.025	**hsa-miR-154-5p***	0.869	1.806
hsa-miR-485-3p****	0.898	5.405	**hsa-miR-4679***	0.863	1.665
hsa-miR-100-3p****	0.861	5.733	hsa-miR-376a-3p*	0.849	1.789
hsa-miR-1185-1-3p**	0.822	2.163	hsa-miR-181c-3p****	0.834	5.444
**hsa-miR-6837-3p******	0.780	4.295	**hsa-miR-539-3p****	0.823	2.666
hsa-miR-510-3p****	0.711	6.281	hsa-miR-27b-5p***	0.707	3.934
**hsa-let-7a-2-3p****	0.646	2.003	**hsa-miR-20a-3p***	0.620	1.707
**hsa-miR-1915-3p***	0.610	1.706	hsa-miR-510-3p***	0.593	3.440
**hsa-miR-4662a-5p*****	0.594	3.488	**hsa-miR-6083****	0.546	2.032
**hsa-miR-376b-3p****	0.579	2.053	hsa-miR-92a-1-5p*	0.525	1.969
**hsa-miR-4441****	0.555	2.082	**hsa-miR-31-5p***	0.485	1.786
**hsa-miR-636****	0.535	2.523	**hsa-miR-4456***	0.484	1.607
hsa-miR-376a-3p***	0.514	3.439	**hsa-miR-6817-3p***	0.453	1.669
**hsa-miR-2117***	0.491	1.973	**hsa-miR-455-5p****	0.451	2.047
hsa-miR-92a-1-5p**	0.489	2.196	**hsa-miR-132-5p***	0.448	1.883
hsa-miR-181c-3p***	0.431	3.894	**hsa-miR-1292-5p***	0.444	1.702
hsa-miR-27b-5p**	0.389	2.197			
**hsa-miR-193b-3p***	0.388	1.804			
hsa-miR-181a-3p**	0.386	2.196			
**hsa-miR-381-3p****	0.320	2.406			
**hsa-miR-134-5p***	0.300	1.992			

**B. Downregulated miRNAs**					
**WT-CFBE Cells**	**Log2FC**	**-log10 (P Value)**	**F508del-CFBE Cells**	**Log2FC**	**-log10 (P Value)**
**hsa-miR-548f-5p***	-1.426	1.982	**hsa-miR-3150b-3p*******	-3.919	4.286
**hsa-miR-4453*******	-0.869	4.838	**hsa-miR-3168*****	-2.704	2.374
**hsa-miR-3157-5p*****	-0.723	2.746	**hsa-miR-1266-5p*******	-2.152	4.383
hsa-miR-3127-5p**	-0.692	2.988	**hsa-miR-505-5p******	-1.736	3.133
**hsa-miR-585-3p*****	-0.632	2.327	**hsa-miR-6808-3p******	-1.628	3.984
**hsa-miR-934*******	-0.604	4.873	**hsa-miR-6087*****	-1.359	2.294
hsa-miR-328-3p**	-0.596	3.445	**hsa-miR-623*****	-1.095	2.172
hsa-miR-330-3p**	-0.520	2.459	**hsa-miR-548ar-5p*****	-1.046	2.172
hsa-miR-1180-3p***	-0.489	3.529	**hsa-miR-152-5p*****	-1.025	2.648
hsa-miR-149-5p****	-0.466	4.897	**hsa-miR-625-3p*****	-1.002	2.464
**hsa-miR-1301-3p*****	-0.382	2.222	**hsa-miR-4449*****	-0.987	2.684
hsa-miR-26b-3p*	-0.378	1.800	hsa-miR-328-3p****	-0.952	4.162
**hsa-miR-2110***	-0.336	1.886	**hsa-miR-1972*****	-0.873	2.092
**hsa-miR-128-3p*****	-0.301	2.759	**hsa-miR-4516*****	-0.786	2.176
hsa-let-7d-3p*	-0.286	1.714	**hsa-miR-619-5p*****	-0.784	2.601
**hsa-miR-30e-3p***	-0.265	1.627	**hsa-miR-4497***	-0.696	1.720
			hsa-miR-3127-5p*	-0.679	1.847
			**hsa-miR-4327***	-0.674	1.912
			hsa-let-7d-3p**	-0.655	2.944
			**hsa-miR-4746-5p***	-0.637	1.863
			hsa-miR-330-3p**	-0.634	2.332
			**hsa-miR-296-3p*****	-0.551	2.036
			**hsa-miR-423-5p*****	-0.512	2.108
			hsa-miR-26b-3p**	-0.483	2.011
			hsa-miR-1180-3p**	-0.444	2.229
			hsa-miR-149-5p*	-0.386	1.899
			**hsa-miR-335-3p***	-0.356	1.716
